# Supplementary material for: The Anticancer Activity Conferred by the Mud Crab Antimicrobial Peptide Scyreprocin through Apoptosis and Membrane Disruption
Source: Int J Mol Sci. 2022 May 14;23(10):5500. doi: 10.3390/ijms23105500 (PMC9142079; doi:10.3390/ijms23105500)
Supplement: Supplementary file 1 [file ijms-23-05500-s001.zip › ijms-1708987-supplementary.pdf]

Supplementary Material

for

The anticancer activity conferred by the mud crab antimicrobial peptide scyreprocin through apoptosis and membrane disruption

Ying Yang[1], Hui-Yun Chen[1,2,3], Hua Hao[1,2,3], Ke-Jian Wang[1,2,3*]

[1] State Key Laboratory of Marine Environmental Science, College of Ocean and Earth Sciences, Xiamen University, Xiamen, 361102, China
[2] State-Province Joint Engineering Laboratory of Marine Bioproducts and Technology, College of Ocean and Earth Sciences, Xiamen University, Xiamen, 361102, China
[3] Fujian Innovation Research Institute for Marine Biological Antimicrobial Peptide Industrial Technology, College of Ocean & Earth Sciences, Xiamen University, Xiamen, Fujian 361102, China
[*] Corresponding author. Tel: +86 592 2184658; E-mail: wkjian@xmu.edu.cn

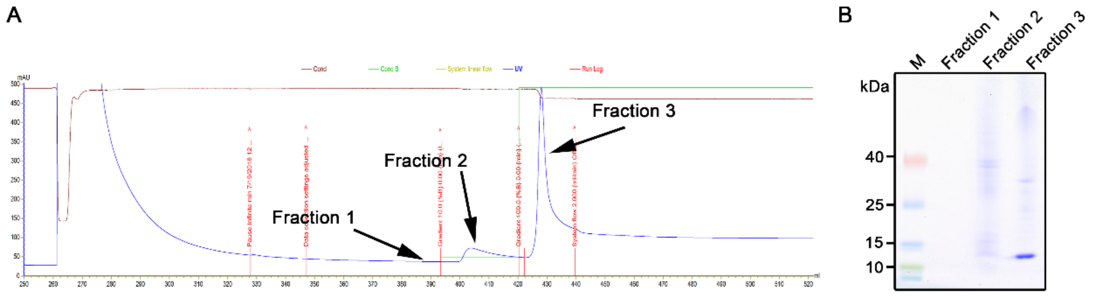

Supplementary Figure S1. Expression and purification of recombinant scyreprocin (rScyreprocin).

(A) Purification of rScyreprocin. Fraction 1, elution of non-targeted proteins; fraction 2, elution of nonspecific binding proteins; fraction 3, elution of rScyreprocin.

(B) Gel analysis of the collected fractions in **(A)**.

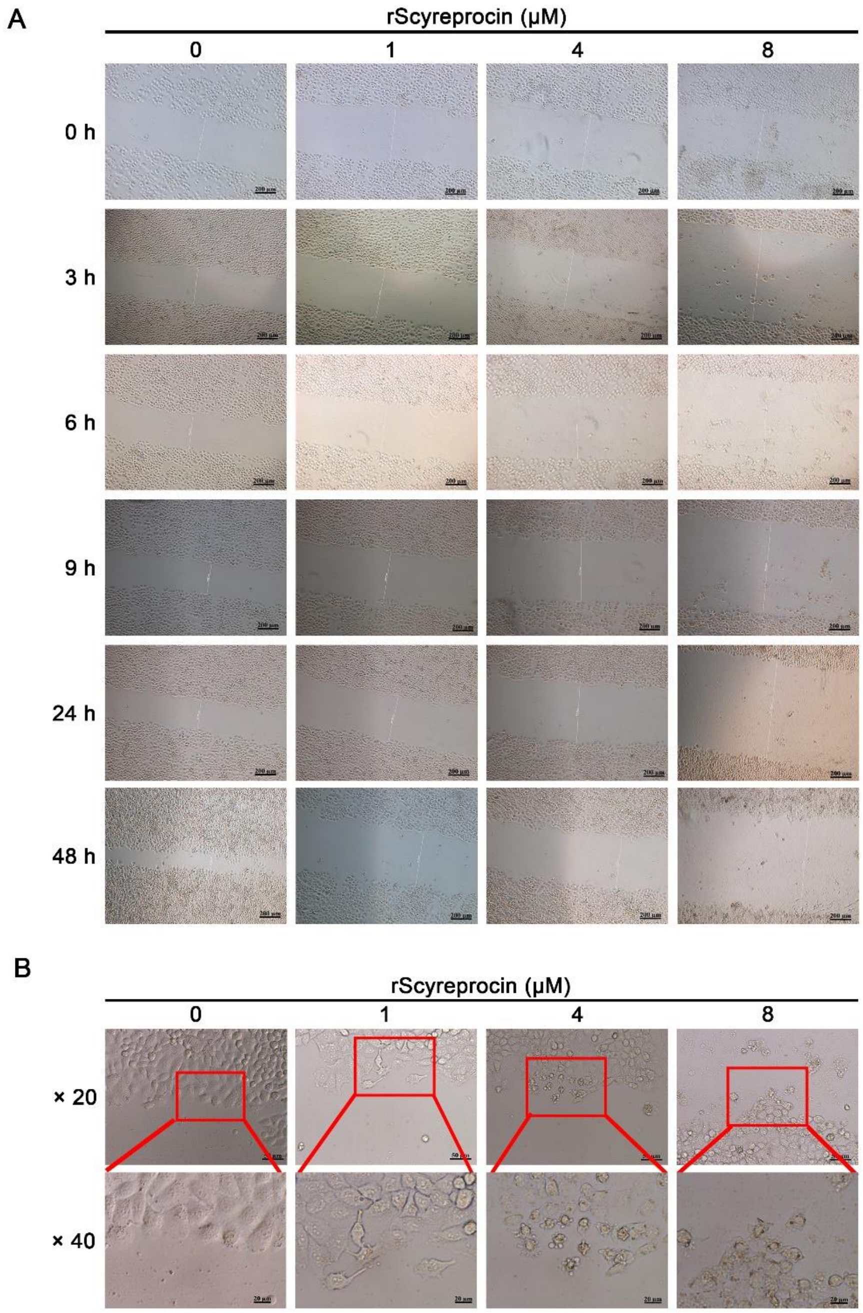

Supplementary Figure S2. Recombinant scyreprocin (rScyreprocin) inhibited migration of NCI-H460 cells.

(A) Wound closure of NCI-H460 cells after rScyreprocin treatment. Scratches were incubated with various concentrations of rScyreprocin, wound closure was observed by an optical microscopy at different time points (scale bar = 200 μm).

(B) Morphological changes of the cells at the edge of the scratches. After treatment with various concentrations of rScyreprocin for 24 h, obvious morphological changes of the cells at the edge of the

scratches were observed compared to that of the control group. Upper panel, edge of scratches (scale bar = 50 μm); lower panel, zoom-in images (scale bar = 20 μm).

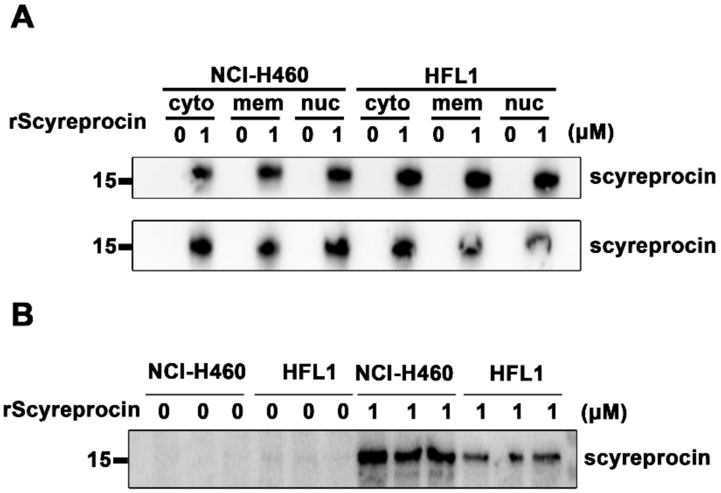

Supplementary Figure S3. Detection of rScyreprocin in different cell fractions.

H460 and HFL1 cells were treated with rScyreprocin (0 and 1 μM) for 24 h. Proteins from cytosolic (cyto), membrane (mem), nucleic (nuc) **(A)** and cytoskeletal fractions **(B)** were extracted and analyzed (4 μg) by Western blot assay using scyreprocin antibody.

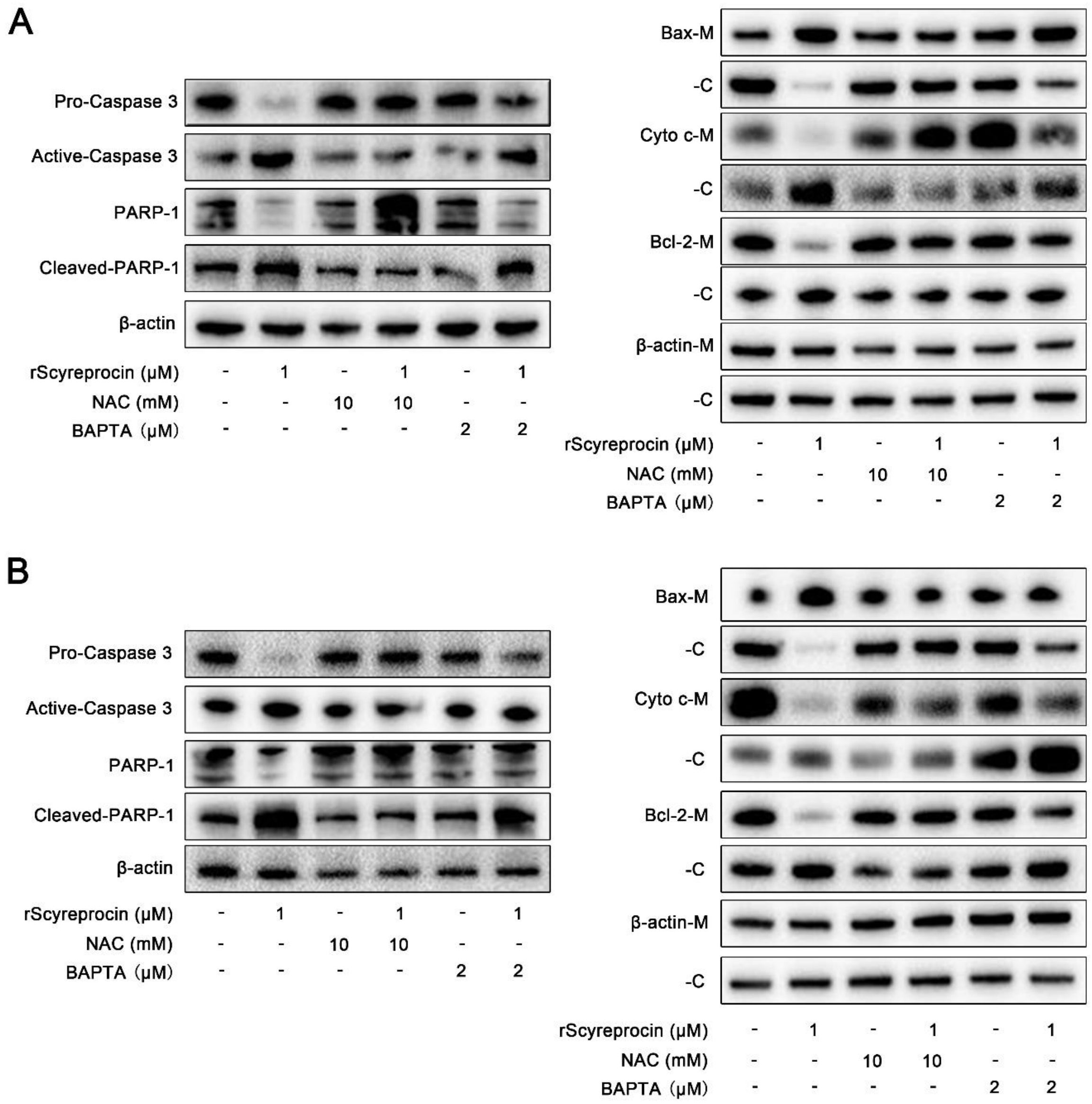

Supplementary Figure S4. Expressions of apoptosis-related proteins in rScyreprocin-treated H460 cells (replicate samples).

Cells were treated with 1μM rScyreprocin for 24 h in the presence or absence of prior 1 h incubation with NAC or BAPTA, respectively. Expressions of apoptosis-related proteins in whole cell lysate (left panel), expressions of Bax, cytochrome c and Bcl-2 in mitochondrial (M) and cytosolic (C) fractions (right panel) were determined.

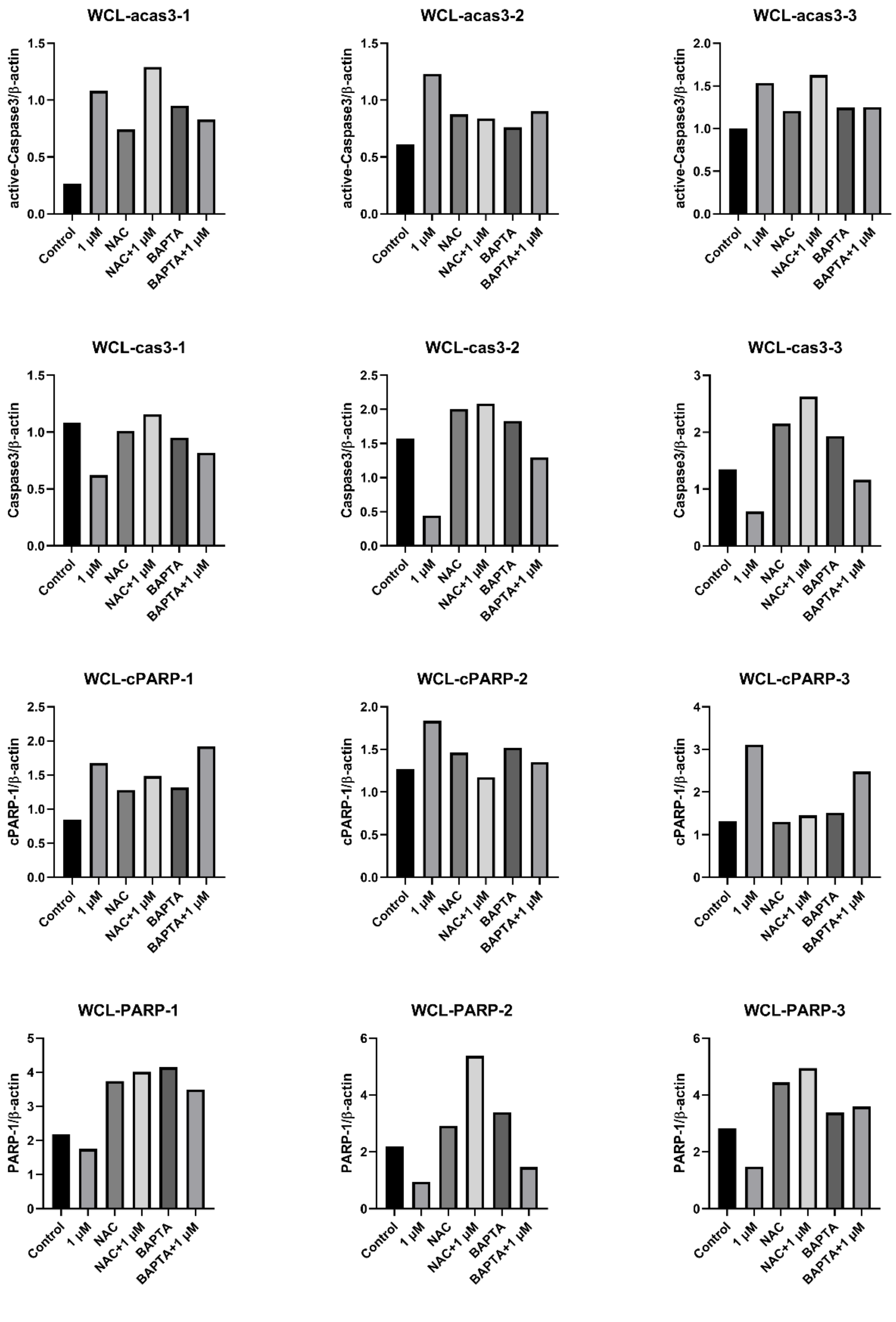

Supplementary Figure S5. Blot quantification of the expression levels of apoptosis-related proteins in rScyreprocin-treated H460 whole cell lysates.

Cells were treated with 1μM rScyreprocin for 24 h in the presence or absence of prior 1 h incubation with NAC or BAPTA, respectively. Blots of apoptosis-related proteins in whole cell lysate in Fig 6G and Fig S5 were quantified and normalized to β-actin using ImageJ software.

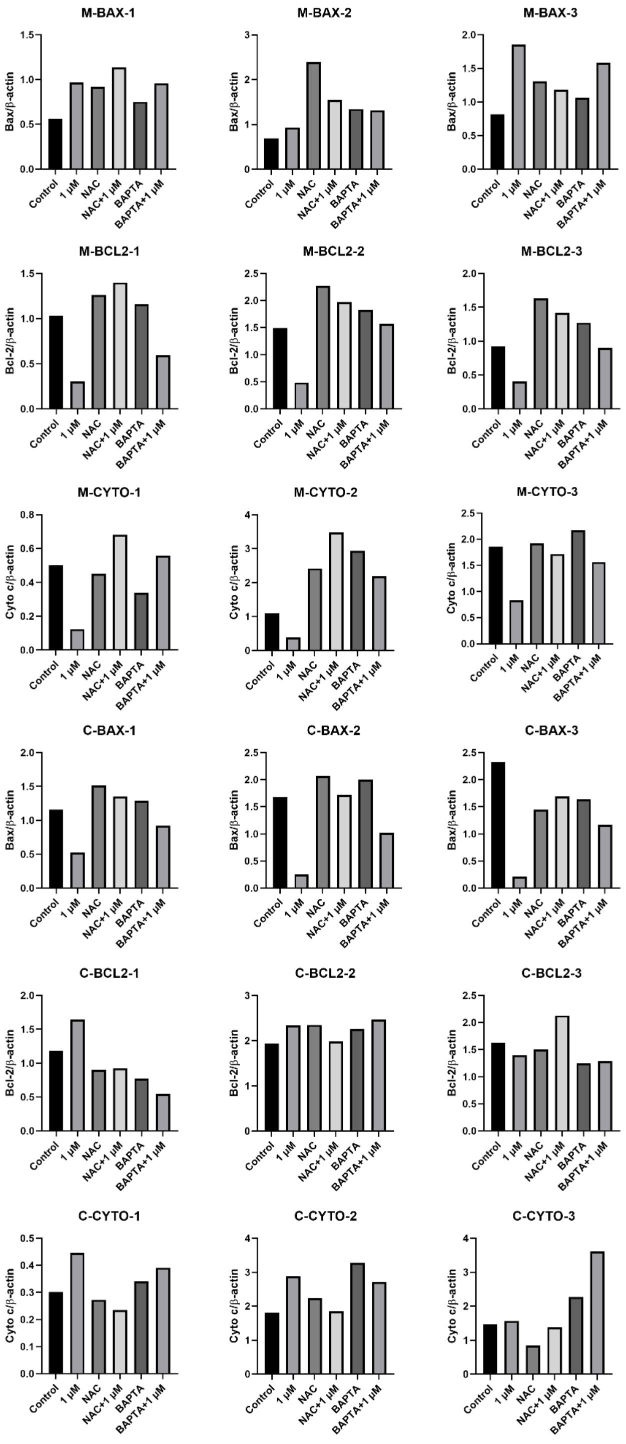

Supplementary Figure S6. Blot quantification of the expression levels of apoptosis-related proteins in mitochondria and cytoplasm of rScyreprocin-treated H460 cells.

Cells were treated with 1μM rScyreprocin for 24 h in the presence or absence of prior 1 h incubation with NAC or BAPTA, respectively. Blots of apoptosis-related proteins in mitochondrial (M) and cytosolic (C) fractions in Fig 6G and Fig S5 were quantified and normalized to β-actin using ImageJ software.

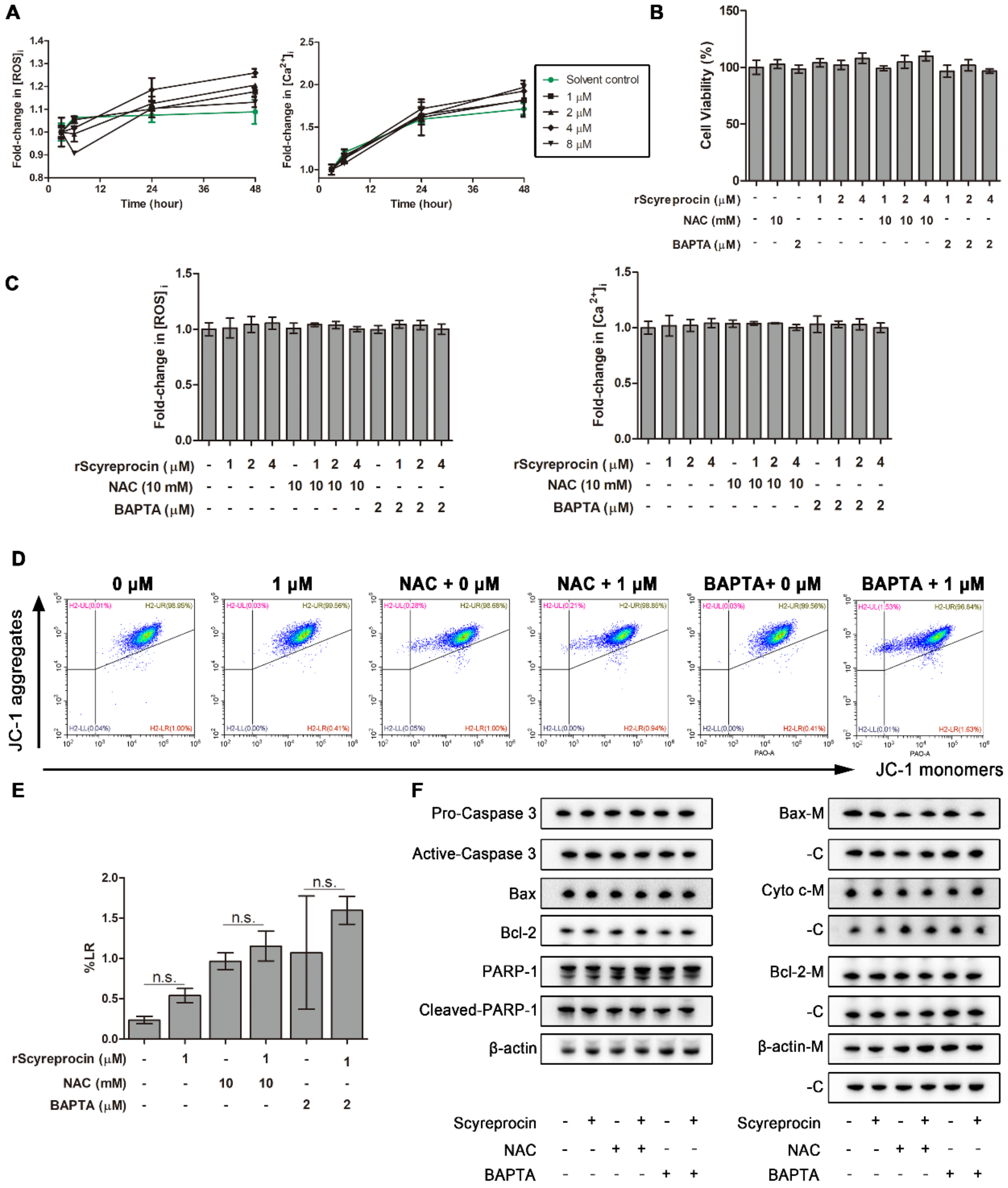

Supplementary Figure S7. Effect of recombinant scyreprocin (rScyreprocin) on HFL1 cells.

(A) Intracellular Ca^{2+} and ROS levels of rScyreprocin-treated HFL1 cells. Cells were treated with rScyreprocin. Intracellular ROS and Ca^{2+} levels were determined at different time points (n = 3).

(B) Inhibitory effect of rScyreprocin on NAC pre-treated HFL1 cells. Cells were treated with rScyreprocin for 24 h in the presence or absence of prior 1 h incubation with 10 mM NAC. The cell viability was evaluated (n = 3).

(C) Effect of rScyreprocin on mitochondrial membrane potential of HFL1 cells. Samples were prepared was described in **(B)**. Cells were subjected for JC-1 staining and analyzed on a flow cytometry (n = 3).

(D) Statistical analysis of the data presented in **(C)**.

(E) Effect of rScyreprocin on intracellular Ca^{2+} and ROS levels of NAC or BAPTA pretreated HFL1 cells. Cells were pretreated with NAC (10 mM) or BAPTA (2 μM) for 1 h prior to rScyreprocin incubation for 24 h (0 and 1 μM), respectively. The intracellular ROS and Ca^{2+} levels were determined, respectively (n = 3).

(F) Levels of apoptosis-related proteins in rScyreprocin-treated HFL1 cells. Cells were treated with 1 μM rScyreprocin for 24 h in the presence or absence of prior 1 h incubation with NAC or BAPTA. Levels of apoptosis-related proteins in whole cell lysate (left panel), levels of Bax, cytochrome c and Bcl-2 in mitochondrial (M) and cytosolic (C) fractions (right panel) were determined (Cells from three wells of 48-well plate were pooled into one for examination).

Data are presented in means ± standard deviations (SD). In **(A)**, data were normalized to the value of solvent control at 3 h. In **(B)**, data were normalized to the control group and analyzed by one-way ANOVA with Tukey post-tests (*$P < 0.05$). In **(D)** and **(F)**, data were analyzed by one-way ANOVA with Tukey post-tests (*$P < 0.05$; n.s., not significant).
